# Association between scene time interval and clinical outcomes according to key Utstein factors in out-of-hospital cardiac arrest

**DOI:** 10.1097/MD.0000000000032351

**Published:** 2022-12-23

**Authors:** Eujene Jung, Hyun Ho Ryu, Young Sun Ro, Sang Do Shin

**Affiliations:** a Department of Emergency Medicine, Chonnam National University Hospital, Gwangju, Korea; b Department of Emergency Medicine, Seoul National University Hospital, Seoul, Korea.

**Keywords:** heart arrest, outcome, scene time interval, Utstein

## Abstract

There is no consensus on the appropriate length of time spent on the scene by emergency medical services. Hence, our study aimed to investigate the association between the scene time interval (STI) and clinical outcomes of out-of-hospital cardiac arrest (OHCA) and determine whether this association is affected by key Utstein factors—witness status, bystander cardiopulmonary resuscitation, and initial electrocardiogram rhythm. This study is a cross-sectional study, using data between 2017 and 2020 from a nationwide, population-based, prospective registry of OHCA. The primary exposure is the STI, which was categorized into 3 groups: short (0 < STI ≤ 12 min), middle (13 ≤ STI ≤ 16 min), long (17 ≤ STI ≤ 30 min). The main outcome was good neurological recovery. Multivariable logistic regression and interaction analyses were performed to estimate the effect of STIs on study outcomes according to key Utstein factors. Witnessed, bystander cardiopulmonary resuscitation, and an initial shockable rhythm were associated with high survival to discharge and good neurological recovery, whereas prolonged STI was associated with low survival to discharge and poor neurological recovery. In patients with witnessed arrest, increased STI caused a more rapid decrease in survival to discharge than in non-witnessed cases (witnessed arrest: 0.56 (0.51–0.62) in middle STI and 0.33 (0.30–0.37) in long STI, non-witnessed arrest: 0.72 (0.61–0.85) in middle STI and 0.53 (0.45–0.62) in long STI. In patients with an initial shockable rhythm, increased STI caused a more rapid decrease in survival to discharge and neurological recovery than in initial non-shockable cases. Longer STIs were associated with poorer OHCA outcomes, and this trend was further emphasized in patients with witnessed OHCA and OHCA with an initial shockable rhythm.

## 1. Introduction

Out-of-hospital cardiac arrest (OHCA) is a time-sensitive life-threatening emergency, with an average global incidence among adults of 55 OHCAs per 100,000 person-years.^[1]^ Despite advances in resuscitation science and public health, the survival-to-discharge rate is only between 3.1% and 20.4% across various geographic settings.^[2]^ Many factors contribute to favorable clinical outcomes in OHCA patients, including but not limited to, a rapid start to cardiopulmonary resuscitation (CPR) and early defibrillation by bystanders, as well as rapid dispatch of emergency medical services (EMS).^[3–5]^ However, there is no consensus on the appropriate length of time spent at the scene by EMS.

In Korea, advanced life support (ALS) procedures provided by EMS providers at the scene are very limited. Moreover, EMS protocols do not allow EMS providers to stop CPR at the scene unless there is return of spontaneous circulation (ROSC), requiring scoop and run to the hospital emergency department (ED) while giving CPR during transport (the “Scoop and Run” model).^[6,7]^ These protocols are very different from those in Europe and North America, where EMS providers continue to perform CPR until getting the ROSC or stop CPR for death declaration on the scene (the “Stay and Treat” model).^[8,9]^ The 2020 American Heart Association guidelines provide no clear recommendation regarding how many CPR cycles are essential for CPR at the scene or how long the EMS provider should stay at the scene.^[10]^

Numerous studies have been conducted on the relationship between key Utstein factors—witness status, bystander CPR, and initial electrocardiogram (ECG) rhythm—as well as clinical outcomes of OHCA patients. In most studies, the survival and neurological outcomes of OHCA were better when the arrest was witnessed, when bystander CPR was performed, or when the initial ECG rhythm was shockable.^[11–14]^ In the US reports using the Cardiac Arrest Registry to Enhance Survival registry, the proportions of the total non-traumatic OHCA cases were 9% for survival-to-hospital discharge cases, 16% for bystander-witnessed cases, and 32% for bystander-witnessed shockable cases.^[15]^

However, based on the information about key Utstein factors before EMS arrival, guidelines and studies on the intensity of on-scene resuscitation and optimal scene time intervals (STIs) are limited. A recent study demonstrated that an STI >20 min is strongly associated with a poor neurological outcome in bystander-witnessed OHCA patients.^[14]^ In another study of refractory OHCA patients with a shockable rhythm, continuing CPR for >15 minutes on the scene was associated with poorer survival and neurological outcomes.^[16]^

We hypothesized that the STI—the duration of stay at the scene to provide CPR—is a key determinant for OHCA outcomes, and the effect of the STI on clinical outcomes following OHCA varies according to key Utstein factors. Therefore, in this study, we analyzed the association between the STI and survival/neurological outcomes following OHCA using data from the National Registry, as part of the Cardiovascular Disease Surveillance (CAVAS) project in Korea, to determine whether this association is affected by key Utstein factors.

## 2. Methods

### 2.1. Study design and setting

This is a cross-sectional study based on a nationwide, population-based, prospective registry of OHCAs, including all patients who were transported by EMS in Korea.

The EMS system of Korea is a single-tiered, government-based system operated by 16 provincial headquarters of the National Fire Agency and covers approximately 50 million individuals living in an area of 100,219 km^2^. There are total of 219 fire stations with approximately 1400 ambulance stations nationwide. In Korea, with the scoop and run EMS system, at least 2 minutes of CPR are performed at the scene and then, the patient is transferred to the hospital regardless of whether ROSC is detected. Emergency medical technicians (EMTs) in Korea are classified into level-1 and level-2 EMTs (comparable to EMT-intermediate and EMT-basic in the USA, respectively). Ambulance crews are usually composed of 3 members (82.4% in 2020), and all ambulances have at least 1 member who is qualified as a level-1 EMT or nurse. Level-1 EMT or nurse can perform advanced airway, mechanical chest compression device, defibrillation, and medication use including epinephrine under direct medical direction at scene and during transport.

In addition, a multitier response (MTR) system was implemented by the National Fire Agency in 2015 across the whole country, consisting of the detection of OHCA by the dispatcher, dispatch of an ambulance and a fire engine in addition to routine dispatch of an ambulance, and the performance of team CPR. Termination of resuscitation at the scene is not permitted. Therefore, all patients with OHCA should be transported to the ED under prehospital CPR except for cases that require withdrawal, such as in the presence of obvious mortality signs or a written do-not-resuscitate order.

All EDs are designated as level 1, 2, or 3 by the government, and there are 460 EDs that are categorized into these 3 levels according to the available resources and capacity such as staffing, equipment, and size of the ED. Level-1 EDs (n = 38) provide 24-hour/365-day emergency care by emergency specialists, level-2 EDs (n = 125) include emergency physicians, and level-3 EDs (n = 239) can include general physicians. All EDs generally perform acute cardiac management and post-cardiac arrest care in accordance with international standard guidelines such as the 2020 American Heart Association guidelines.

Most of the level-1 and some level-2 hospitals perform post-cardiac arrest care such as targeted temperature management, coronary reperfusion therapy, and extracorporeal membrane oxygenation.

### 2.2. Data source

This research was part of a national population-based study, the CAVAS project, which has been conducted since 2006 in collaboration with the National Emergency Management Agency and the Korea Centers for Disease Control (CDC) of the Korean government. The CAVAS project has constructed a nationwide EMS-assessed OHCA cohort, for which demographic and Utstein information was drawn from the EMS run sheet. This cohort was followed by a hospital medical record review for hospital resuscitation and post-resuscitation care and outcomes.

The data quality management committee (QMC) was charged with maintaining data quality via a data quality management protocol throughout the study project. The QMC is composed of a cardiologist, emergency physicians, an epidemiologist, medical record review experts, and statistical experts. Monthly QMC meetings provided feedback to all medical record reviewers to ensure data quality.^[17,18]^

### 2.3. Study participants

The data were extracted between January 2017 and December 2020. All adults >18 years and with presumed cardiac etiology were included. Patients were excluded, if ROSC is achieved at scene; if resuscitation was not attempted by EMS; for whom there was no information of STI; or if the recorded STI was <0 minutes or >30 minutes.

Patients having an arrest of cardiac etiology were identified by medical record review. We presumed non-cardiac etiology if the medical record described the cause of cardiac arrest definitely as trauma, hanging, asphyxia, drowning, burn, and poisoning. Without definite non-cardiac etiology, we presumed the arrest to be of cardiac etiology.

### 2.4. Outcome measures

The primary outcome was good neurological recovery, which was defined as having a cerebral performance categories score of 1 (good recovery) or 2 (mild disability). The cerebral performance category score was determined by medical record reviewers employed at the Korea CDC and was based on discharge summary abstracts. The secondary outcome was survival to discharge, which was determined on the basis of the medical record and discharge summary by medical record reviewers of the Korea CDC.

### 2.5. Variables

The main exposure variable was STI, which was defined from the time of arrival of EMS at the scene to the time of departure from the scene. The time interval was measured using an automatic tracking system connected to the dispatcher center. The STI was categorized into 3 groups: short (0 < STI ≤ 12 min), middle (13 ≤ STI ≤ 16 min), and long (17 ≤ STI ≤ 30 min). The interaction variables were key Utstein variables—witness status, bystander CPR, and the initial ECG rhythm.^[19]^

We collected data about characteristics of the patients, including age, sex, community urbanization (metropolitan or non-metropolitan), and comorbidities such as hypertension, diabetes mellitus, and heart disease. We also collected data from the EMS run sheet about arrest characteristics, including the place of arrest; prehospital time-related variables, including response time interval, which was defined as the time interval from call to ambulance arrival at the scene, and transport time interval (TTI), which was defined as the time interval to departure from the scene; multitier response; number of EMS providers; EMS defibrillation; epinephrine use by EMS; and level of ED.

### 2.6. Statistical analysis

Demographic findings and outcomes were examined on the basis of STI and key Utstein factors. Continuous variables were compared using the Wilcoxon rank sum test and categorical variables were compared using the chi-square test.

A multivariable logistic regression analysis was performed to estimate the effect of STI on neurological recovery and survival-to-hospital discharge for all eligible study subjects and calculate the adjusted odds ratios and 95% confidence intervals after adjusting for potential confounders. We introduced an interaction term for STI and key Utstein factors into the multivariable logistic regression model to estimate the effect of STI changes according to key Utstein factors on the study outcomes. We tested the multicollinearity between co-variables in the model. All statistical analyses were performed using SAS-version 9.4 software (SAS Institute Inc., Cary, NC).

## 3. Results

### 3.1. Demographic findings

**Figure 1.**
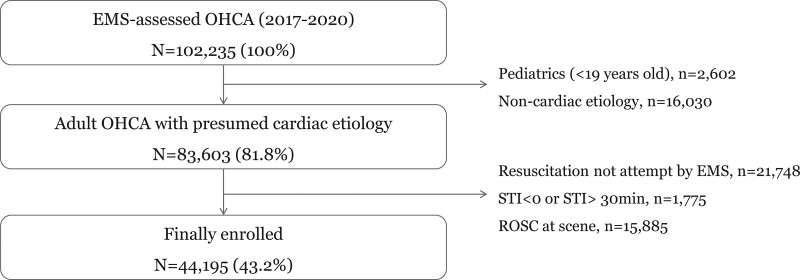
Study populations.

A total of 44,195 adult OHCA patients with presumed cardiac etiology were included in this analysis (Fig. 1). The characteristics grouped according to STI (short: 0–12, middle: 13–16, long: 17–30) are shown in Table 1. In the group with short STI, witnessed arrest and an initial shockable rhythm were more common and airway management was not frequently performed, whereas in the group with long STIs, a multitier response was frequently implemented. With prolonged STIs, prehospital ROSC, survival to discharge, and neurological recovery all decreased significantly.

**Table 1:** Characteristics of the study population according to scene time interval.

Variables	All	Scene time interval			
		Short	Middle	Long	
	N (%)	N (%)	N (%)	N (%)	*P* value
All	44,195 (100.0)	16,335 (100.0)	12,402 (100.0)	15,458 (100.0)	
Scene time interval, median (IQR)	14 (11–18)	10 (8–11)	14 (13–15)	20 (18–23)	<.01
Age					<.01
18–64	13,560 (30.7)	5041 (30.9)	3667 (29.6)	4852 (31.4)	
65	30,635 (69.3)	11,294 (69.1)	8735 (70.4)	10,606 (68.6)	
Sex, female	16,358 (37.0)	6201 (38.0)	4617 (37.2)	5540 (35.8)	
Metropolis, yes	17,016 (38.5)	5979 (36.6)	5092 (41.1)	5945 (38.5)	<.01
Comorbidity					
Hypertension	16,248 (36.8)	5869 (35.9)	4622 (37.3)	5757 (37.2)	.02
Diabetes mellitus	10,815 (24.5)	3787 (23.2)	3094 (24.9)	3934 (25.4)	
Heart disease	8551 (19.3)	3174 (19.4)	2385 (19.2)	2992 (19.4)	.91
Place of arrest					<.01
Private	27,308 (61.8)	9897 (60.6)	7717 (62.2)	9694 (62.7)	
Public	16,887 (38.2)	6438 (39.4)	4685 (37.8)	5764 (37.3)	
Witness, yes	21,972 (49.7)	9062 (55.5)	5730 (46.2)	7180 (46.4)	
Bystander CPR, yes	25,438 (57.6)	8614 (52.7)	7838 (63.2)	8986 (58.1)	
Initial ECG, shockable	6787 (15.4)	2756 (16.9)	1796 (14.5)	2235 (14.5)	
Response time interval					<.01
0–5 min	10,046 (19.3)	3754 (19.4)	2773 (19.2)	3519 (19.4)	
6–10 min	23,996 (80.7)	8472 (80.6)	6854 (80.8)	8670 (80.6)	
11 min	10,153 (0.0)	4109 (0.0)	2775 (0.0)	3269 (0.0)	
Transport time interval					<.01
0–4 min	11,786 (26.7)	3927 (24.0)	3473 (28.0)	4386 (28.4)	
5–8 min	15,305 (34.6)	5317 (32.5)	4497 (36.3)	5491 (35.5)	
9 min	17,104 (38.7)	7091 (43.4)	4432 (35.7)	5581 (36.1)	
Multi-tier response, yes	33,506 (75.8)	9907 (60.6)	10,158 (81.9)	13,441 (87.0)	
EMT number					<.01
3 persons	29,688 (67.2)	10,869 (66.5)	8612 (69.4)	10,207 (66.0)	
1 or 2 persons	14,507 (32.8)	5466 (33.5)	3790 (30.6)	5251 (34.0)	
Airway management					<.01
ETI or SGA	19,168 (43.4)	6622 (40.5)	5601 (45.2)	6945 (44.9)	
BVM	23,378 (52.9)	8567 (52.4)	6543 (52.8)	8268 (53.5)	
None	1649 (3.7)	1146 (7.0)	258 (2.1)	245 (1.6)	
EMS defibrillation, yes	9319 (21.1)	3462 (21.2)	2443 (19.7)	3414 (22.1)	<.01
ED level					
Level 1	10,311 (23.3)	3643 (22.3)	2788 (22.5)	3880 (25.1)	<.01
Level 2	20,785 (47.0)	7236 (44.3)	6074 (49.0)	7475 (48.4)	<.01
Level 3	13,099 (29.6)	5456 (33.4)	3540 (28.5)	4103 (26.5)	<.01
Hospital treatment					
TTM	1591 (3.6)	636 (3.9)	425 (3.4)	530 (3.4)	.04
Reperfusion	3296 (7.5)	1615 (9.9)	852 (6.9)	829 (5.4)	<.01
ECMO	562 (1.3)	226 (1.4)	136 (1.1)	200 (1.3)	.09
Study outcomes					
Prehospital ROSC	16,518 (37.4)	6963 (42.6)	4326 (34.9)	5229 (33.8)	<.01
Survival to discharge	4184 (9.5)	2257 (13.8)	1039 (8.4)	888 (5.7)	<.01
Good neurological recovery	2853 (6.5)	1611 (9.9)	690 (5.6)	552 (3.6)	<.01

CPR = cardiopulmonary resuscitation, ECG = electrocardiogram, ECMO = extracorporeal membrane oxygenation, ED = emergency department, EMS = emergency medical service, EMT = emergency medical technician, ETI = endotracheal intubation, IQR = interquartile range, ROSC = return of spontaneous circulation, SGA = supraglottic airway, TTM = targeted temperature management.

The characteristics of the OHCA patients according to key Utstein factors—witness status, bystander CPR, and initial ECG rhythm—are shown in Table 2. More cases of an initial shockable rhythm were observed in witnessed arrest, and among patients with witnessed arrest and an initial shockable rhythm, there were more patients with short STI of <12 minutes (41.2% and 40.6%, respectively). A short STI was also common in patients with non-bystander CPR (41.2%). Among patients with witnessed arrest, bystander CPR, and an initial shockable rhythm, clinical outcomes of prehospital ROSC, survival to discharge, and good neurological recovery were significantly better.

**Table 2:** Characteristics of the study population according to key Utstein factors.

Variables	All	Witness status			Bystander CPR			Initial ECG rhythm		
		Witness (+)	Witness (−)		Bystander CPR (+)	Bystander CPR (−)		Shockable	Non-shockable	
	N (%)	N (%)	N (%)	*P* value	N (%)	N (%)	*P* value	N (%)	N (%)	*P* value
All	44,195 (100.0)	21,972 (100.0)	22,223 (100.0)		25,438 (100.0)	18,757 (100.0)		6787 (100.0)	37,408 (100.0)	
Scene time interval, median (IQR)	14 (11–18)	14 (10–18)	15 (11–19)	<.01	15 (11–18)	14 (10–18)	<0.01	14 (10–18)	14 (11–18)	<0.01
Age				<.01			<0.01			<0.01
18–64	13,560 (30.7)	7008 (31.9)	6552 (29.5)		7934 (31.2)	5626 (30.0)		3926 (57.8)	9634 (25.8)	
65	30,635 (69.3)	14,964 (68.1)	15,671 (70.5)		17,504 (68.8)	13,131 (70.0)		2861 (42.2)	27,774 (74.2)	
Sex, female	16,358 (37.0)	8120 (37.0)	8238 (37.1)	.8	9858 (38.8)	6500 (34.7)	<0.01	1292 (19.0)	15,066 (40.3)	<0.01
Metropolis, yes	17,016 (38.5)	8509 (38.7)	8507 (38.3)	.33	9464 (37.2)	7552 (40.3)	<0.01	2835 (41.8)	14,181 (37.9)	<0.01
Comorbidity										
Hypertension	16,248 (36.8)	8393 (38.2)	7855 (35.3)	<.01	9450 (37.1)	6798 (36.2)	0.05	2353 (34.7)	13,895 (37.1)	<0.01
Diabetes mellitus	10,815 (24.5)	5490 (25.0)	5325 (24.0)	.01	6173 (24.3)	4642 (24.7)	0.24	1345 (19.8)	9470 (25.3)	<0.01
Heart disese	8551 (19.3)	4520 (20.6)	4031 (18.1)	<.01	4918 (19.3)	3633 (19.4)	0.93	1852 (27.3)	6699 (17.9)	<0.01
Place of arrest				<.01			<0.01			<0.01
Private	27,308 (61.8)	13,295 (60.5)	14,013 (63.1)		15,226 (59.9)	12,082 (64.4)		2842 (41.9)	24,466 (65.4)	
Public	16,887 (38.2)	8677 (39.5)	8210 (36.9)		10,212 (40.1)	6675 (35.6)		3945 (58.1)	12,942 (34.6)	
Witness, yes	21,972 (49.7)	21,972 (100.0)	(0.0)	<.01	12,646 (49.7)	9326 (49.7)	0.99	4870 (71.8)	17,102 (45.7)	<0.01
Bystander CPR, yes	25,438 (57.6)	12,646 (57.6)	12,792 (57.6)	.99	25,438 (100.0)	(0.0)	<0.01	4585 (67.6)	20,853 (55.7)	<0.01
Initial ECG, shockable	6787 (15.4)	4870 (22.2)	1917 (8.6)	<.01	4585 (18.0)	2202 (11.7)	<0.01	6787 (100.0)	(0.0)	<0.01
Response time interval				<.01			<0.01			<0.01
0–5 min	10,046 (22.7)	4922 (22.4)	5124 (23.1)		5839 (23.0)	4207 (22.4)		1692 (24.9)	8354 (22.3)	
6–10 min	23,996 (54.3)	11,826 (53.8)	12,170 (54.8)		13,913 (54.7)	10,083 (53.8)		3712 (54.7)	20,284 (54.2)	
11 min	10,153 (23.0)	5224 (23.8)	4929 (22.2)		5686 (22.4)	4467 (23.8)		1383 (20.4)	8770 (23.4)	
Scene time interval				<.01			<0.01			<0.01
Short, 0–12 min	16,335 (37.0)	9062 (41.2)	7273 (32.7)		8614 (33.9)	7721 (41.2)		2756 (40.6)	13,579 (36.3)	
Middle, 13–16 min	12,402 (28.1)	5730 (26.1)	6672 (30.0)		7838 (30.8)	4564 (24.3)		1796 (26.5)	10,606 (28.4)	
Long, 17–30 min	15,458 (35.0)	7180 (32.7)	8278 (37.2)		8986 (35.3)	6472 (34.5)		2235 (32.9)	13,223 (35.3)	
Transport time interval				<.01			<0.01			<0.01
0–4 min	11,786 (26.7)	5452 (24.8)	6334 (28.5)		6848 (26.9)	4938 (26.3)		1733 (25.5)	10,053 (26.9)	
5–8 min	15,305 (34.6)	7495 (34.1)	7810 (35.1)		8941 (35.1)	6364 (33.9)		2229 (32.8)	13,076 (35.0)	
9 min	17,104 (38.7)	9025 (41.1)	8079 (36.4)		9649 (37.9)	7455 (39.7)		2825 (41.6)	14,279 (38.2)	
Multi-tier response, yes	33,506 (75.8)	15,427 (70.2)	18,079 (81.4)	<.01	21,714 (85.4)	11,792 (62.9)	<0.01	5209 (76.7)	28,297 (75.6)	0.05
EMT number				<.01			0.94			<0.01
3 persons	29,688 (67.2)	14,508 (66.0)	15,180 (68.3)		17,084 (67.2)	12,604 (67.2)		4673 (68.9)	25,015 (66.9)	
1 or 2 persons	14,507 (32.8)	7464 (34.0)	7043 (31.7)		8354 (32.8)	6153 (32.8)		2114 (31.1)	12,393 (33.1)	
Airway management				<.01			<0.01			<0.01
ETI or SGA	19,168 (43.4)	9269 (42.2)	9899 (44.5)		11,541 (45.4)	7627 (40.7)		2959 (43.6)	16,209 (43.3)	
BVM	23,378 (52.9)	11,539 (52.5)	11,839 (53.3)		13,387 (52.6)	9991 (53.3)		3530 (52.0)	19,848 (53.1)	
None	1649 (3.7)	1164 (5.3)	485 (2.2)		510 (2.0)	1139 (6.1)		298 (4.4)	1351 (3.6)	
EMS defibrillation, yes	9319 (21.1)	6014 (27.4)	3305 (14.9)	<.01	5993 (23.6)	3326 (17.7)	<0.01	6487 (95.6)	2832 (7.6)	<0.01
ED level				<.01			0.07			<0.01
Level 1	10,311 (23.3)	5645 (25.7)	4666 (21.0)		5835 (22.9)	4476 (23.9)		2011 (29.6)	8300 (22.2)	
Level 2	20,785 (47.0)	10,533 (47.9)	10,252 (46.1)		12,005 (47.2)	8780 (46.8)		3308 (48.7)	17,477 (46.7)	
Level 3	13,099 (29.6)	5794 (26.4)	7305 (32.9)		7598 (29.9)	5501 (29.3)		1468 (21.6)	11,631 (31.1)	
Hospital treatment										
TTM	1591 (3.6)	1064 (4.8)	527 (2.4)	<.01	890 (3.5)	701 (3.7)	0.18	710 (10.5)	881 (2.4)	<0.01
Reperfusion	3296 (7.5)	2523 (11.5)	773 (3.5)	<.01	2060 (8.1)	1236 (6.6)	<0.01	2376 (35.0)	920 (2.5)	<0.01
ECMO	562 (1.3)	395 (1.8)	167 (0.8)	<.01	310 (1.2)	252 (1.3)	0.25	331 (4.9)	231 (0.6)	<0.01
Study outcomes										
Prehospital ROSC	16,518 (37.4)	10,852 (49.4)	5666 (25.5)	<.01	8996 (35.4)	7522 (40.1)	<0.01	4452 (65.6)	12,066 (32.3)	<0.01
Survival to discharge	4184 (9.5)	3306 (15.0)	878 (4.0)	<.01	2500 (9.8)	1684 (9.0)	<0.01	2734 (40.3)	1450 (3.9)	<0.01
Good neurological recovery	2853 (6.5)	2362 (10.8)	491 (2.2)	<.01	1881 (7.4)	972 (5.2)	<0.01	2283 (33.6)	570 (1.5)	<0.01

CPR = cardiopulmonary resuscitation, ECG = electrocardiogram, ECMO = extracorporeal membrane oxygenation, ED = emergency department, EMS = emergency medical service, EMT = emergency medical technician, ETI = endotracheal intubation, IQR = interquartile range, ROSC = return of spontaneous circulation, SGA = supraglottic airway, TTM = targeted temperature management.

### 3.2. Main outcomes

The results of the multivariable logistic regression model are presented as adjusted odds ratios (95% confidence intervals) for key Utstein factors and STIs in Table 3. There was a strong association between the following Utstein factors and survival to discharge and good neurological recovery: witnessed arrest (4.13 [3.81–4.48]) and (5.08 [4.58–5.63]), bystander CPR (1.09 [1.01–1.17] and 2.26 [1.61–2.98]), an initial shockable rhythm (10.26 [9.46–11.12] and 17.41 [15.66–19.38]), respectively. A prolonged STI was associated with poor survival to discharge (0.60 [0.55–0.65] in the middle STI and 0.37 [0.34–0.41] in the long STI groups), as well as poor neurological recovery (0.55 [0.49–0.60] in the middle STI, 0.32 [0.29–0.36] in the long STI groups).

**Table 3:** Multivariable logistic regression analysis on study outcomes by scene time interval and key Utstein factors.

Study outcomes	Total	Outcome		Model 1	Model 2
	N	n	%	AOR (95% CI)	AOR (95% CI)
Survival to discharge					
Total	44,195	4184	9.5		
Witness (−)	22,223	878	4.0	Reference	Reference
Witness (+)	21,972	3306	15.0	4.26 (3.93–4.61)	4.13 (3.81–4.48)
Bystander CPR (−)	18,757	1684	9.0	Reference	Reference
Bystander CPR (+)	25,438	2500	9.8	1.19 (1.11–1.27)	1.09 (1.01–1.17)
Non-shockable	37,408	1450	3.9	Reference	Reference
Shockable	6787	2734	40.3	11.27 (10.41–12.20)	10.26 (9.46–11.12)
STI, short	16,335	2257	13.8	Reference	Reference
STI, middle	12,402	1039	8.4	0.60 (0.55–0.65)	0.60 (0.55–0.65)
STI, long	15,458	888	5.7	0.38 (0.35–0.41)	0.37 (0.34–0.41)
Good neurological recovery					
Total	44,195	2853	6.5		
Witness (−)	22,223	491	2.2	Reference	Reference
Witness (+)	21,972	2362	10.8	5.29 (4.78–5.86)	5.08 (4.58–5.63)
Bystander CPR (−)	18,757	972	5.2	Reference	Reference
Bystander CPR (+)	25,438	1881	7.4	1.65 (1.51–1.80)	2.26 (1.61–2.98)
Non-shockable	37,408	570	1.5	Reference	Reference
Shockable	6787	2283	33.6	19.39 (17.46–21.53)	17.42 (15.66–19.38)
STI, short	16,335	1611	9.9	Reference	Reference
STI, middle	12,402	690	5.6	0.54 (0.49–0.60)	0.55 (0.49–0.60)
STI, long	15,458	552	3.6	0.32 (0.29–0.35)	0.32 (0.29–0.36)

Model 1 adjusted for age, sex, urbanization; Model 2 adjusted for variables in Model 1 and hypertension, diabetes mellitus, heart disease, and location of arrest. AOR = adjusted odds ratio, CI = confidence interval, CPR = cardiopulmonary resuscitation, STI = scene time interval.

### 3.3. Interaction analysis

We analyzed the interactions between STI and the key Utstein factors with respect to survival and neurological outcomes (Table 4). In patients with witnessed arrest, prolonged STIs caused a more rapid decrease in survival to discharge than in non-witnessed cases (witnessed arrest: 0.56 [0.51–0.62] in middle STI and 0.33 [0.30–0.37] in long STI, non-witnessed arrest: 0.72 [0.61–0.85] in middle STI and 0.53 [0.45–0.62] in long STI). In patients with an initial shockable rhythm, prolonged STIs caused a more rapid decrease in survival to discharge than cases with an initial non-shockable rhythm (shockable rhythm: 0.54 [0.47–0.61] in middle STI and 0.30 [0.27–0.35] in the long STI, non-shockable rhythm: 0.63 [0.55–0.71] in middle STI and 0.41 [0.36–0.47] in the long STI). Similarly, poor neurological recovery was also more rapid in cases with an initial non-shockable rhythm (shockable rhythm: 0.41 [0.33–0.51] in middle STI and 0.27 [0.24–0.32] in the long STI, non-shockable rhythm: 0.57 [0.50–0.65] in middle STI and 0.33 [0.26–0.41] in the long STI). The STI did not have any interaction effect with bystander CPR.

**Table 4:** Interaction analysis between the scene time interval and key Utstein factors.

	Scene time interval			
	Short	Middle	Long	
	AOR (95% CI)	AOR (95% CI)	AOR (95% CI)	*P* for interaction
Survival to discharge				<.01
Witness (−)	Reference	0.72 (0.61–0.85)	0.53 (0.45–0.62)	
Witness (+)	Reference	0.56 (0.51–0.62)	0.33 (0.30–0.37)	
Bystander CPR (−)	Reference	0.55 (0.48–0.63)	0.37 (0.32–0.43)	
Bystander CPR (+)	Reference	0.63 (0.57–0.70)	0.38 (0.34–0.42)	
Non-shockable	Reference	0.63 (0.55–0.71)	0.41 (0.36–0.47)	
Shockable	Reference	0.54 (0.47–0.61)	0.30 (0.27–0.35)	
Good neurological recovery				.20
Witness (−)	Reference	0.63 (0.53–0.81)	0.40 (0.31–0.50)	
Witness (+)	Reference	0.52 (0.46–0.58)	0.30 (0.27–0.34)	
Bystander CPR (−)	Reference	0.49 (0.41–0.59)	0.33 (0.27–0.40)	
Bystander CPR (+)	Reference	0.57 (0.51–0.65)	0.32 (0.28–0.36)	
Non-shockable	Reference	0.57 (0.50–0.65)	0.33 (0.26–0.41)	
Shockable	Reference	0.41 (0.33–0.51)	0.27 (0.24–0.32)	

AOR = adjusted odds ratio, CI = confidence interval, CPR = cardiopulmonary resuscitation.

## 4. Discussion

Our study, which investigated the effect of STI on OHCA outcomes according to the key Utstein factors—witness status, bystander CPR, and initial ECG rhythm—found that the odds of survival to discharge and good neurological recovery decreased with increasing STI. Further, the odds of survival outcomes decreased more rapidly as the STI increased in witnessed OHCA compared to non-witnessed OHCA. In patients with an initial shockable rhythm, the survival and neurological outcomes deteriorated sharply for STIs of >17 minutes compared to patients with an initial non-shockable rhythm.

The main results of our study that the clinical outcomes of OHCA worsen with increasing STIs are consistent with previous data reporting a direct association between STIs and the likelihood of poor neurologic outcomes, providing further evidence that conventional prehospital resuscitation strategies may have a definable, time-dependent therapeutic window.^[20–23]^ Recent retrospective analysis of national OHCA data from the USA found that longer STIs are strongly associated with poor neurological recovery in bystander-witnessed OHCA patients.^[24]^ However, a study of OHCA patients without prehospital ROSC categorized STI into 4 groups: short (0 < STI < 4 min), middle (4 ≤ STI < 8 min), long (8 ≤ STI < 12 min), and very long (12 ≤ STI < 60 min) and found that the middle STI category was associated with the highest odds of good neurological recovery.^[22]^

In addition, many studies on factors affecting the relationship between STI and OHCA outcomes have also been reported. A study of OHCA patients without prehospital ROSC showed that a longer TTI adversely affected the likelihood of good neurological recovery, and this negative effect was more prominent in the short STI group. Another study on refractory OHCA patients with a shockable rhythm showed that on-scene resuscitation for >15 minutes was associated with poorer survival and neurological outcomes.

The above studies provide evidence that there may be a basis for considering the initiation of transfer along with the expected TTI. In this study, we divided STIs into 3 groups: short (0 < STI ≤ 12 min), middle (13 ≤ STI ≤ 16 min), and long (17 ≤ STI ≤ 30) and found that the longer the STI, the worse the OHCA outcome. We also analyzed whether this trend differs according to witness status, bystander CPR, and the initial ECG rhythm—key Utstein factors that can be identified at the time of EMS arrival.

This study is the first to study whether the effect of STI on OHCA outcome may differ depending on the key Utstein factors—witness status, bystander CPR, and initial ECG rhythm.

In our study subjects, the proportion of short STI (0–12 min) was higher in witnessed arrest, and that of the EMS defibrillation was almost double that of non-witnessed arrest (27.4% vs 14.9%). The decrease in the odds of survival to discharge in the group with longer STIs was independent of whether the arrest was witnessed or not. However, in the case of witnessed arrest, the decrease in survival to discharge with increased STI was significantly steeper than that observed in non-witnessed arrest.

Consistent with the results of previous studies^[14,25]^ when the initial ECG measured by the EMS at the scene was shockable, the survival to discharge and good neurological recovery were overwhelmingly high. However, in the analysis of the interaction with STIs, the OHCA outcome deteriorated more rapidly as the STI increased in the patients with an initial shockable rhythm compared to those with a non-shockable rhythm. It is not clear why witnessed arrest or an initial shockable rhythm—both reported to be associated with favorable OHCA outcomes in previous studies—respond more rapidly to the adverse effects of an increase in STI than non-witnessed arrest or initial non-shockable rhythm. Possible explanations could be that because the no-flow time or low-flow time can be expected to be relatively long in the case of non-witnessed arrest or an initial non-shockable rhythm, improving oxygenation and circulation through ALS at the scene rather than rushing transport to the hospital may improve the OHCA outcomes. Thus, in patients with an initial shockable rhythm, it may be important to receive time-dependent post-resuscitation care through rapid transport to the hospital.

The results of our study do not imply that the ultimate goal should be to reduce STI by excluding essential ALS, although in the case of witnessed arrest or an initial shockable rhythm, efforts to reduce STI may be necessary by suppressing factors that delay STI rather than focusing on essential ALS.

Our results that a prolonged STI is associated with lower odds of survival to discharge and poor neurological recovery, especially prominent in OHCA cases of witnessed arrest and those with a shockable rhythm, may provide a theoretical basis for determining the appropriate scene resuscitation strategies by confirming the key Utstein factors—witness status, bystander CPR, and an initial ECG rhythm.

Our study has several limitations. First, the study design was not a randomized, controlled trial. There may be significant potential biases that were not controlled. Second, since on-scene EMS protocol of Korea just requires at least 2 minutes of CPR, there is a possibility of STI selection bias depending on the on-scene situation or judgments of paramedics or medical director. Third, our findings cannot be generalized to other EMS systems, because the study examined the intermediate service level of EMS. Fourth, we could not directly measure the quality of CPR and the treatments provided by EMS at scene. Fifth, advanced airway management and defibrillation are mainly performed at the scene, but sometimes they are performed during transport. However, we did not measure the objective time of the procedures or how many procedures attempts were made at the scene. Lastly, information regarding clinical outcomes obtained by the medical record reviewers from electronic medical records could represent a measurement bias because we did not test the reliability of the measurement.

## 5. Conclusion

In our study using a nationwide OHCA registry, longer STIs were associated with poor OHCA outcomes, and this trend was further emphasized in patients with witnessed OHCA and OHCA with an initial shockable rhythm. Our study can help decision-making by EMS providers that determines scene resuscitation based on the key Utstein factors—witness status, bystander CPR, and an initial ECG rhythm.

## Author contributions

**Conceptualization:** Eujene Jung, Hyun Ho Ryu, Sang Do Shin.

**Data curation:** Eujene Jung.

**Formal analysis:** Eujene Jung, Young Sun Ro, Sang Do Shin, Hyun Ho Ryu.

**Funding acquisition:** Sang Do Shin.

**Investigation:** Eujene Jung, Young Sun Ro, Sang Do Shin.

**Methodology:** Young Sun Ro, Sang Do Shin.

**Project administration:** Young Sun Ro, Sang Do Shin.

**Resources:** Sang Do Shin.

**Software:** Eujene Jung.

**Supervision:** Hyun Ho Ryu.

**Validation:** Eujene Jung, Hyun Ho Ryu.

**Visualization:** Young Sun Ro

**Writing – original draft:** Eujene Jung.

**Writing – review & editing:** Hyun Ho Ryu.
